# Prokaryotic homologs of Argonaute proteins are predicted to function as key components of a novel system of defense against mobile genetic elements

**DOI:** 10.1186/1745-6150-4-29

**Published:** 2009-08-25

**Authors:** Kira S Makarova, Yuri I Wolf, John van der Oost, Eugene V Koonin

**Affiliations:** 1 National Center for Biotechnology Information, NLM, National Institutes of Health, Bethesda, Maryland 20894, USA; 2 Laboratory of Microbiology, Department of Agrotechnology and Food Sciences, Wageningen University, Dreijenplein 10, 6703 HB Wageningen, Netherlands

## Abstract

**Background:**

In eukaryotes, RNA interference (RNAi) is a major mechanism of defense against viruses and transposable elements as well of regulating translation of endogenous mRNAs. The RNAi systems recognize the target RNA molecules via small guide RNAs that are completely or partially complementary to a region of the target. Key components of the RNAi systems are proteins of the Argonaute-PIWI family some of which function as slicers, the nucleases that cleave the target RNA that is base-paired to a guide RNA. Numerous prokaryotes possess the CRISPR-associated system (CASS) of defense against phages and plasmids that is, in part, mechanistically analogous but not homologous to eukaryotic RNAi systems. Many prokaryotes also encode homologs of Argonaute-PIWI proteins but their functions remain unknown.

**Results:**

We present a detailed analysis of Argonaute-PIWI protein sequences and the genomic neighborhoods of the respective genes in prokaryotes. Whereas eukaryotic Ago/PIWI proteins always contain PAZ (oligonucleotide binding) and PIWI (active or inactivated nuclease) domains, the prokaryotic Argonaute homologs (pAgos) fall into two major groups in which the PAZ domain is either present or absent. The monophyly of each group is supported by a phylogenetic analysis of the conserved PIWI-domains. Almost all pAgos that lack a PAZ domain appear to be inactivated, and the respective genes are associated with a variety of predicted nucleases in putative operons. An additional, uncharacterized domain that is fused to various nucleases appears to be a unique signature of operons encoding the short (lacking PAZ) pAgo form. By contrast, almost all PAZ-domain containing pAgos are predicted to be active nucleases. Some proteins of this group (e.g., that from *Aquifex aeolicus*) have been experimentally shown to possess nuclease activity, and are not typically associated with genes for other (putative) nucleases. Given these observations, the apparent extensive horizontal transfer of pAgo genes, and their common, statistically significant over-representation in genomic neighborhoods enriched in genes encoding proteins involved in the defense against phages and/or plasmids, we hypothesize that pAgos are key components of a novel class of defense systems. The PAZ-domain containing pAgos are predicted to directly destroy virus or plasmid nucleic acids via their nuclease activity, whereas the apparently inactivated, PAZ-lacking pAgos could be structural subunits of protein complexes that contain, as active moieties, the putative nucleases that we predict to be co-expressed with these pAgos. All these nucleases are predicted to be DNA endonucleases, so it seems most probable that the putative novel phage/plasmid-defense system targets phage DNA rather than mRNAs. Given that in eukaryotic RNAi systems, the PAZ domain binds a guide RNA and positions it on the complementary region of the target, we further speculate that pAgos function on a similar principle (the guide being either DNA or RNA), and that the uncharacterized domain found in putative operons with the short forms of pAgos is a functional substitute for the PAZ domain.

**Conclusion:**

The hypothesis that pAgos are key components of a novel prokaryotic immune system that employs guide RNA or DNA molecules to degrade nucleic acids of invading mobile elements implies a functional analogy with the prokaryotic CASS and a direct evolutionary connection with eukaryotic RNAi. The predictions of the hypothesis including both the activities of pAgos and those of the associated endonucleases are readily amenable to experimental tests.

**Reviewers:**

This article was reviewed by Daniel Haft, Martijn Huynen, and Chris Ponting.

## Background

The discovery of elaborate and versatile systems of RNA-mediated gene silencing in eukaryotes is one of the pivotal advances in biology of the last decade [1-5]. There are three major, distinct forms of regulatory small RNAs involved in eukaryotic gene silencing: small interfering (si) RNAs, micro (mi) RNAs, and PIWI-associated (pi) RNA (previously referred to as rasiRNA) [6]. The siRNAs are derived from double-stranded RNAs of viruses and transposable elements, which are processed by Dicer, one of the essential components of the RNA-Induced Silencing Complexes (RISCs) [7-11]. Dicer cleaves long dsRNA molecules into short, 21–22 nucleotide duplexes which are subsequently unwound and the guide strand is loaded on another crucial component of RISC, the Argonaute (Ago) slicer nuclease. The Ago-siRNA complex then binds to the target mRNA which is cleaved by the PIWI domain of Argonaute (Ago), after which the mRNA fragments are released and the RISC-siRNA catalytic complex is recycled [9,12-14].

Variant, paralogous Dicers and Argonautes are involved in the mechanisms of the other classes of small RNA such as miRNA and piRNA [14]. Unlike the siRNAs, 21–25 nt-long miRNAs are encoded in eukaryotic genomes and are either perfectly (in plants) or imperfectly (in animals) complementary to sequences in the 3'-untranslated regions of specific endogenous mRNAs [12]. Base-pairing of miRNAs with the target mRNAs, which is mediated by a distinct form of RISC, results either in RNA cleavage or in down-regulation of translation without cleavage [8]. Evidence is rapidly accumulating that numerous of miRNAs in animals and plants are major players in development regulation and chromatin remodeling [3].

Dicer and Argonaute are the core components of RISCs. Dicer is a multi-domain protein that typically consists of a DEXD/H-type helicase domain fused with an RNA-binding PAZ domain, two RNAse III domains, and in some cases a dsRNA-binding domain [14]. The Argonaute protein is composed of four domains including the PAZ RNA-binding domain and the PIWI family exonuclease, and performs the slicer function [9,12,13]. Both Dicer and Argonaute are represented by variable numbers of paralogs in eukaryotes, and different paralogs are included in RISCs with distinct functions [9,12,13].

Prokaryotes possess apparent functional counterparts to the miRNA system, that is, regulation of bacterial gene expression by small antisense RNAs. The best characterized of these pathways employ the RNA-binding protein Hfq for small RNA presentation and RNAse E for target degradation [15-17]. *Escherichia coli *appears to encode ~60 microRNA genes [18,19], and comparable numbers of expressed, small antisense RNAs have been detected in the archaea *Archaeoglobus fulgidus *[20] and *Sulfolobus solfataricus *[8] suggesting an important role of this regulatory mechanism in prokaryotic physiology. In addition, small antisense RNAs have been shown to regulate plasmid replication and to kill plasmid-free bacterial cells by silencing specific plasmid genes [21].

The recently discovered major prokaryotic phage/plasmid defense system, the CRISPR associated system (CASS) [[22,23], Waters, 2009 #566], also relies on guide RNA that apparently targets invader DNA [24]. The hallmark of the CASS is that this system encompasses a still poorly understood mechanism for integrating fragments of bacteriophage DNA into a specific site within the CRISPR repeat cassette; at least in part, integration of these fragments is probably mediated by the Cas1 proteins that has been predicted [22,25] and more recently experimentally demonstrated to possess DNAse activity [26]. The unique, phage/plasmid-specific CRISPR inserts are then transcribed and processed to guide RNAs that are directed to the target DNA by the Cascade complex which (in *Escherichia coli *K12) consists of 5 Cas proteins and seems to a be a functional analog of the RISC [27]. Despite general functional analogies, the molecular mechanisms of CASS and eukaryotic RNAi are distinct, and the protein components of the two systems are not homologous [22,28].

Many archaea and bacteria do encode homologs of the major protein components of eukaryotic RNAi, in particular, Argonaute-PIWI family proteins, and the helicase and RNAse III domains of Dicer although the fusion of these domains in a single protein appears to be a eukaryotic signature [29]. The crystal structures of Argonaute homologs from two thermophilic bacteria [30,31] and two archaea [32,33] have been solved, and the structures appear to be very similar to those of eukaryotic Argonautes [34]. However the functions of the prokaryotic Argonaute homologs (hereinafter pAgo) remain obscure, despite the *in vitro *demonstration of the RNAse H-like ribonuclease activity (cleavage of RNA in a DNA/RNA duplex) of the pAgos from the bacteria *Aquifex aeolicus *[35] and *Thermus thermophilus *[36].

Here, we apply comparative genomics and in-depth computational analysis of Argonaute-PIWI family proteins and other proteins that are typically encoded in their genomic neighborhoods to predict the biological functions of pAgo. We present a hypothesis that the prokaryotic Argonautes are key components of a novel class of virus/plasmid defense systems.

## Results and Discussion

### Prokaryotic Argonaute homologs belong to two major groups based on the presence or absence of the PAZ domain

To identify all prokaryotic Argonaute homologs, we performed a PSI-BLAST search against the NCBI non-redundant protein sequence database using the PIWI domain (the most highly conserved domain in the Argonaute family proteins) sequence from the *Thermus thermophilus *HB27 pAgo (TT_P0026, pdb: 3DLB containing; PIWI domain sequences in amino acid positions 415–685). The search was run until convergence (after the 3^rd ^iteration) and resulted in the identification of 100 sequences, some of which were fragmented or truncated proteins; additional searches started with some of the detected proteins showed that this sequence set represents the full complement of PIWI-domain proteins (pAgo) encoded in currently available prokaryotic genomes. For more detailed analysis, we selected 85 sequences from 80 genomes (the genomes of the bacteria *Parvularcula bermudensis *HTCC2503 and *Halorubrum lacusprofundi *ATCC 49239 encode three pAgo proteins each, and the genome of *Acidobacterium capsulatum *ATCC 51196 encodes two pAgos) (see Additional File 1).

**Figure 1:**
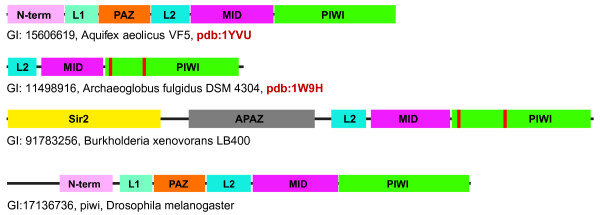
**Domain architecture variation in homologs of Argonaute from prokaryotes (pAgos) and eukaryotes (Ago)**. Structural domains (N-term, L1, PAZ, L2, Mid, PIWI) are projected from the tertiary structure of AaAgo (pdb: 1YVU[35]). Red bars show the inactivated catalytic sites of PIWI domain. Sir2, predicted Sir2 family nuclease domain. APAZ, a domain identified in this work that is associated with pAgos. The domains are shown roughly to scale.

Comparative sequence analysis of the identified pAgos showed that the conserved, alignable region shared by all these sequences approximately corresponded to the L2, Mid and PIWI domains, as inferred from the crystal structures of the pAgos from the hyperthermophilic bacterium *Aquifex aeolicus *(AaAgo; pdb: 1YVU[35]), *Thermus thermophilus *(TtAgo; pdb 3DLB[31,36]), as well as the archaea *Pyrococcus furiosus *(PfAgo; pdb 1Z25[33]) and *Archaeoglobus fulgidus *(AfAgo; pdb: 1W9H[37]) (Figure 1; see also Additional File 2). In addition to the three conserved domains, both pAgos whose structures have been solved contain an N-terminal domain, an L1 domain, and a PAZ domain that, as in eukaryotic Argonaute, binds the 3' end of a siRNA guide and positions the middle of siRNA guide bound to the target mRNA in the catalytic pocket of the PIWI nuclease [32-34]. However, among the identified pAgos, more than half lack the N-terminal, L1 and PAZ domains although several instead contain an N-terminal fusion with predicted nucleases of the Sir2 family (Figure 1 and see details below).

### PIWI domain is inactivated in numerous pAgos

**Figure 2:**
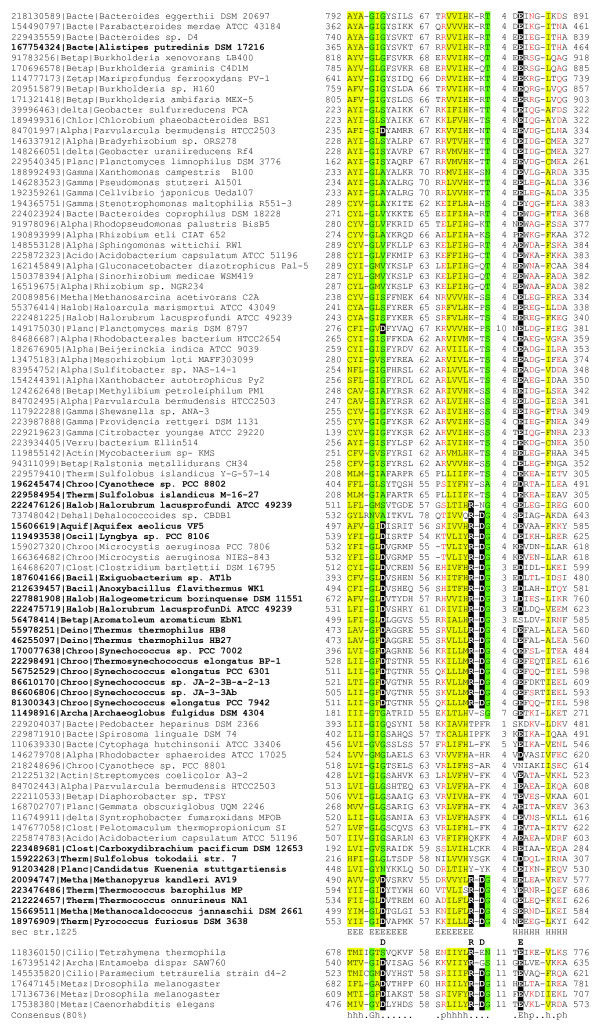
**Prokaryotic PIWI-domains: predicted active nucleases and apparently inactivated forms**. The multiple sequence alignment includes the core motifs of PIWI domains encompassing the amino acid residues that comprise the (D/E)-(D/E)XK active site. The sequences are denoted by their GI numbers and species names. The positions of the first and the last residues of the aligned region in the corresponding protein are indicated for each sequence. The numbers within the alignment represent poorly conserved inserts that are not shown. The catalytic residues of the D-RD-EXK active site are shown in reverse shading and shown underneath the secondary structure, which corresponds to the solved structure for Pf-Ago (PDB: 1Z25); 'H' indicates α-helix, 'E' indicates extended conformation (β-strand). Sequence identifiers for pAgos that are not associated with other proteins in putative operons are highlighted in bold. The coloring is based on the consensus shown underneath the alignment; 'h' indicates hydrophobic residues (WFYMLIVACTH), 'p' indicates polar residues (EDKRNQHTS), 's' indicates small residues (ACDGNPSTV).

The PIWI domain of Argonaute proteins belongs to the RNAse H fold and shares the divalent cation-binding motif DDE (aspartate, aspartate, glutamate) involved in catalysis with many other nucleases that cleave both RNA and DNA [38]. The two aspartates are essential for the slicer activity of eukaryotic Argonautes whereas the third catalytic residue can be glutamate, histidine, aspartate or lysine [34]. Another conserved feature of Argonautes is the presence of a basic residue (in most instances, arginine) that is located in the catalytic site [35]. Some eukaryotic Argonaute proteins appear to be inactive (hence denoted non-slicer Argonautes), especially, in nematodes [34]. Apparently, non-slicer Argonautes interfere with translation through binding rather than cleavage of mRNA [39]. Examination of the multiple alignment of the catalytic cores of prokaryotic PIWI domains strongly suggests that the majority of these domains are inactivated as indicated by the replacement of two or all three acidic residues required for catalysis; this apparent abrogation of the nuclease activity is particularly common in those pAgo proteins that lack the PAZ domain (Figure 2).

The AfAgo protein, which does not contain a PAZ domain, also lacks the catalytic aspartates but has been shown to bind dsRNA [32,40]. Structural analysis of AfAgo complexed with a siRNA-like duplex showed that in this protein a Cd^2+ ^ion bound to the carboxy-terminal carboxylate and several amino acid residues in the middle (MID) domain are involved in the recognition of the unpaired 5' nucleotide of siRNA [32,40]. In contrast, a structural and biochemical study of AaAgo, which contains the PAZ domain and the conserved catalytic residues, showed that this protein is an active RNAse H with a preference for a DNA/RNA hybrid as a substrate, suggesting that some pAgos employ small guide DNA molecules to cleave mRNA [35]. The detailed study of the *Thermus thermophilus *pAgo corroborated the findings on AaAgo by revealing the details of interactions with the 5'-phosphorylated 21-base DNA guide strand and the DNA-guided RNA cleavage by this protein [31,36].

### Phylogenetic analysis of the Argonaute family suggests extensive horizontal gene transfer in prokaryotes

**Figure 3:**
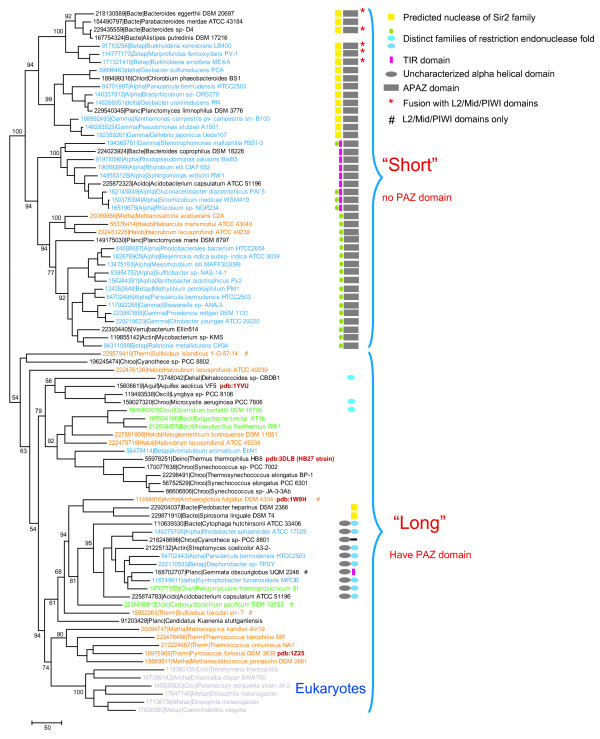
**Phylogenetic analysis of PIWI-domains and organization of the predicted pAgo operons**. The ML tree is rooted between the (predominantly) PAZ-domain-containing and PAZ-domain- lacking branches. The RELL bootstrap values are indicated (%) for selected major branches. Color code: gray, Eukaryota; orange, Archaea; blue, Proteobacteria, green, Firmicutes; black, other lineages of bacteria. Each organism is denoted by the full systematic name and the Gene Identifier (GI) number. The PDB ID is indicated for those sequences for which tertiary structure is solved. Sequences of short PIWI proteins (that have lost N-terminal part including PAZ domain) but belong to the branch that consists mostly of full size sequences are indicated by "#" symbol. For those PIWI-domain proteins that are associated with genes encoding a nuclease domain, the domain architectures of the pAgo-associated proteins are shown.

We constructed a phylogenetic tree of the PIWI domains from all the detected pAgos (after excluding sequences that were fragmented or truncated due to poor annotation) and a subset of eukaryotic Argonautes (Figure 3). The majority of the PIWI domains from pAgos that lack a PAZ domain form a distinct clade although a few of these short forms cluster within the other clade that consists mostly of full-size, PAZ-containing pAgos. Within the latter clade, the short proteins do not form a distinct group (Figure 3), suggesting the N-terminal part of pAgo was lost independently in several lineages. Consistent with the similarity of domain architectures and with the results of previous analyses [29], eukaryotic Argonautes belong to a well-supported clade together with a distinct subset of archaeal pAgos; in particular the structurally characterized *Pyrococcus furiosus *protein, that is considered to be the model for Argonaute functioning in eukaryotes [33]. Other archaeal proteins are scattered in the tree, suggesting multiple horizontal gene transfers (HGT) between bacteria and archaea (Figure 3). Despite the existence of several small lineage-specific groups (alpha proteobacteria, gamma proteobacteria, bacteroides and cyanobacteria), the results of our phylogenetic analysis strongly suggest that pAgo genes mostly disseminated by HGT; the patchy distribution of these genes makes it unlikely that they perform indispensible functions in any bacteria or archaea (Figure 3).

### The pAgos are contextually linked to at least three distinct families of predicted nucleases

We further examined the genomic context of the pAgo genes; analysis of genomic context has been established as a powerful approach for prediction of the biological functions of prokaryotic genes using the "guilt by association" principle [41-43]. In many cases, these genes form potential operons with a variety of genes encoding uncharacterized proteins (neighbor genes were predicted to be encoded in a potential operon with pAgos if they were located upstream or downstream of the respective pAgo gene on the same DNA strand and if the intergenic distances in such an array of co-directional genes were shorter than 100 nt; see Additional File 1). We performed an in-depth analysis of the sequences of the proteins encoded in the genes co-localized with pAgos using PSI-BLAST, HHpred and CDD search (see Methods). This analysis resulted in the identification of four protein families that are predicted to be co-expressed and thus functionally linked with the pAgos.

**Figure 4:**
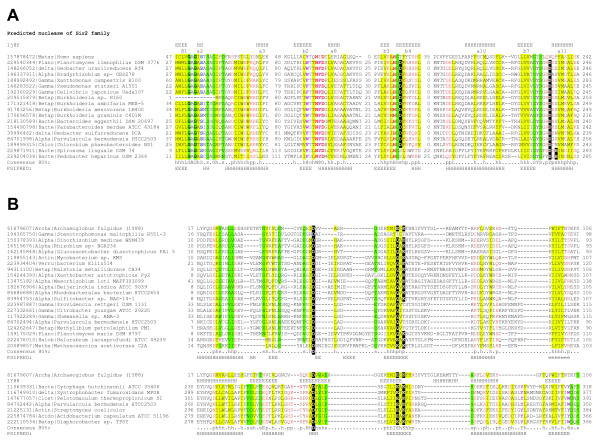
**Multiple alignment of predicted nuclease domains found in the genomic neighborhoods of pAgo genes**. A. Predicted nucleases of the Sir2 family. Numbering of the secondary structure elements corresponds that those reported for PDB: 1j8f[51]. B. (D/E)-(D/E)XK family nucleases. The designations are as in Figure 1. Additional coloring is 'o', hydroxyl-group containing residues (ST); '@', aromatic residues (YWF).

The first family is typified by the xccb100_3097 protein from *Xanthomonas campestris *B100, the only protein among the pAgo neighbors that, in the current sequence databases, is annotated as a "putative Sir2-family regulator" rather than a "hypothetical protein". Indeed, CDD search detected statistically significant similarity between the N-terminal domain of this protein and the SIR2 domain (cl00195, E-value = 5 × 10^-5^). The Sir2 proteins, also known as sirtuins, are a well characterized family of NAD^+^-dependent histone deacetylases in eukaryotes where they play key roles in the regulation of gene silencing, DNA repair, metabolic enzymes, and life span [44-47]. Representatives of this family also have been identified in both bacteria and archaea, and the structures of several Sir2 family proteins have been solved [48,49]. So far all experimentally characterized Sir2 family proteins have been shown to possess protein deacetylase activity [48]. However, a distinct family of prokaryotic sirtuins is associated with DNA-pumping ATPases of the FtsK-HerA family [50]. Because in numerous other instances the FtsK-like ATPases are associated with known nucleases, both functionally and in terms of the operon structure, it was hypothesized that this particular family of sirtuins could function as nucleases, and a conserved DxH motif was implicated in the predicted nuclease activity [50]. The majority of the xccb100_3097-like proteins contain only one of these residues, namely, the aspartate in the loop between strand 7 and helix 11 (according to the crystal structure of human Sirt2 histone deacetylase, pdb: 1j8f[51]) but instead have an additional aspartate in the strand 2 that is conserved within this family(Figure 4A). Similarly to Sir2 proteins associated with the FtsK-like ATPases, xccb100_3097-like proteins lack the Zn-ribbon insert between strand 4 and helix 10 that is characteristic of most sirtuins, but retain all NAD^+^-binding site residues, suggesting that these proteins are active enzymes (Figure 4A).

For the C-terminal domain of xccb100_3097, we failed to detect any statistically significant similarities to known domains using CDD search or HHpred. However, PSI-BLAST search with the xccb100_3097 used as a query revealed many homologs with similar domain architectures, all of which are associated with pAgos in putative operons; moreover, several multidomain proteins (eg. GIs: 91783256, 218130589, 229435559) comprise fusions of xccb100_3097-like and PIWI domains (see the alignment of this domain in Additional File 3).

The second family of PIWI-associated proteins is typified by the mlr6203 (GI: 13475182) protein from *Mesorhizobium loti*. The HHpred search convincingly shows that the N-terminal domain of these proteins belongs to the Mrr family of restriction endonucleases, with the hallmark (D/E)-(D/E)XK active site [52,53] (for example, the best hit is to pdb: 2ost, homing endonuclease from *Synechocystis sp*., E-value = 0.04; followed by a hit to pfam04471, Restriction endonuclease, E-value = 0.04). All experimentally characterized superfamily representatives are site-specific endonucleases that cleave dsDNA and possess an enormous variety of recognition sites [52-54]. The active site residues are conserved in all mlr6203 homologs (Figure 4B), so this domain probably is an active DNA endonuclease. As with the xccb100_3097 family proteins, no similarity to the C-terminal domain of the mlr6203 was detected in CDD and HHpred searches. However, the PSI-BLAST search identified 17 homologous proteins with the same domain architecture and predicted operon organization (see Additional File 1).

A typical representative of the third family is RHECIAT_PB0000019 (GI: 190894000) from *Rhizobium etli*. This protein contains an N-terminal TIR domain that was easily detected by HHpred (the best hit is to pdb: 2js7, TIR domain of myeloid differentiation primary response protein MYD88 from human, E-value of 1.1 × 10^-30^). The TIR domain mediates protein-protein interactions and belongs to the STIR superfamily that includes mostly eukaryotic proteins involved in diverse signaling pathways as well as a variety of poorly characterized multidomain proteins from bacteria and archaea with large genomes (that also have been implicated in transcription regulation and signaling [55-57]). Notably, TIR domains play important roles in disease and stress resistance in plants [58]. Similarly, in mammals, TIR-domains are key components of the immune system-based antimicrobial and antiviral response, and the programmed cell death (PCD) system [59,60]. Analysis of domain architectures led to the hypothesis that prokaryotic TIR-domain proteins also could be involved in PCD [61]. All closely related homologs of the RHECIAT_PB0000019 protein contain the TIR domain (see Additional File 3), whereas several proteins in this family (e.g. GI: 162145848) also contain an additional N-terminal domain that belongs to the PD-(D/E)XK nuclease superfamily (a vast assemblage of nucleases that includes, among others, the restriction endonucleases) with all catalytic residues typically conserved (Figure 4B). The C-terminal domain of these proteins is not similar to any known domain, but does show a weak sequence similarity (with statistical significance difficult to demonstrate) to the C-terminal domain of the mlr6203-like family. Considering similar sizes of the corresponding domains in both families and, most importantly, the genomic association with predicted nucleases and pAgos, we strongly suspect that these domains are homologous; examination of their multiple alignment indeed shows several distinct, conserved motifs (see Additional File 3). The predicted secondary structure indicates that this is a globular domain, however, the pattern of amino acid residue conservation does not seem to suggest an enzymatic function. Given that the proteins containing this domain are found exclusively in the same neighborhoods with pAgos that lack the PAZ domain, it is tempting to speculate that this uncharacterized domain is functionally analogous to the PAZ domain, that is, involved in binding a guide nucleic acid molecule (hereinafter we refer to this domain as APAZ, after Analog of PAZ).

The fourth family of pAgo-associated proteins is linked to full-size, PAZ-domain-containing Argonaute homologs and can be typified by the protein PTH_0722 (GI: 147677057) from *Pelotomaculum thermopropionicum*. This protein contains a C-terminal domain that belongs to the PD-(D/E)XK nuclease superfamily (HHPred detects similarity to SfsA: Sugar fermentation stimulation protein, which contains a PD-(D/E)XK nuclease domain, with E-value = 0.022) and contains all the catalytic residues (Figure 4B); this putative nuclease is clearly distinct from and only very distantly related to the restriction endonuclease domain of the mlr6203-like family proteins. The N-terminal domain of this protein does not show similarity to any characterized domains, has a predicted predominantly α-helical structure and is present only in close homologs of PTH_0722 (see Additional File 4). In the GobsU_24486 protein of *Gemmata obscuriglobus*, the nuclease domain is replaced by the apparently functionally unrelated SEFIR domain of the STIR superfamily, that is only distantly related to the TIR domain, but is also involved in various signaling pathways [57].

Several other genomic neighbors of pAgos are worth mentioning (Figure 3). Two genes that encode PAZ-domain-containing but, apparently, inactivated pAgos (in the bacteria *Pedobacter heparinus *and *Spirosoma lingual*) are associated with predicted Sir2 family nucleases (Figure 4A). Furthermore, three long forms of pAgos (one inactivated, in the bacterium *Dehalococcoides *sp, and two apparently active ones in *Microcystis aeruginosa *and *Clostridium bartletti*) are associated with PD-(D/E)XK nucleases of a distinct subfamily related to Cas4 (COG1468), which is mostly represented within CASS [22]. Most conspicuously, as noticed previously, in the archaeon *Methanopyrus kandleri*, the pAgo is encoded within an operon that otherwise encodes components of the CASS [22].

A potentially important pattern revealed by this analysis of the genomic context of prokaryotic PIWI-domain proteins is that, almost without exception, pAgos with an apparently inactivated catalytic PIWI domain are associated with a predicted nuclease in a putative operon (Figures 2, 3 and see Additional File 1). This observation suggests the possibility of functional complementarity between the nuclease activity of PIWI domains of pAgos and other nucleases, in particular, homologs of restriction endonucleases (see discussion below).

### Statistical analysis of the genomic neighborhoods of pAgos reveals a significant link to phage resistance systems

Considering (i) the central role of Argonaute proteins in siRNA-based antiviral response in eukaryotes, (ii) the contextual links between pAgos and nucleases (in particular, restriction endonucleases) that are involved in phage/plasmid defense in prokaryotes, and (iii) links to the TIR domain that also functions in antimicrobial response in eukaryotes, it is tempting to hypothesize that an important if not the principal function of the pAgos has to do with phage defense (or, more generally, defense against viruses, plasmids, and other mobile elements). Phage defense systems in prokaryotes are notably prone to HGT (the CASS being the prime showcase), and phylogenetic analysis of the pAgos clearly indicates that HGT shapes the evolution of pAgo-encoding genes as well (Figure 3). In addition, phage defense systems are often encoded in genomic islands [62]. Therefore we sought to statistically test the hypothesis that pAgo genes are non-randomly associated with known phage resistance genes in prokaryotic genomes. To this end, we identified 4 classes of phage defense systems (some of which are also involved in a broader range of stress response reactions) in a representative set of 45 prokaryotic genomes and computed the fractions of these genes throughout the genomes and in the vicinity of pAgo genes (see Methods for details). The Fisher Omnibus test [63,64] reveals a statistically highly significant enrichment of the pAgo genomic neighborhoods (see Methods for details) for different combinations of 4 classes of phage defense genes used as a target set (Table 1). As a control, we performed the same analysis for pAgo genes and typical components of the bacterial mobilome including transposases and various phage-derived genes; no statistically significant association was found between pAgos and these mobile genes (p = 0.63; see Additional Files 5 and 6).

**Table 1:** Results of the Fisher Omnibus test for the genomic association of pAgo genes with four classes of phage defense/stress response systems

RM	ABI	CASS	TA	Combined p-value
+	+	-	-	5.1 × 10^-7^

+	+	-	+	2.9 × 10^-13^

+	+	+	-	5.8 × 10^-10^

+	+	+	+	4.6 × 10^-16^

RM, Restriction-modification related COGs; ABI, abortive infection related COGs; CASS, CASS-associated systems; TA, toxin-antitoxin systems related COGs. The phage defense systems that were included in the target genes combination in each of the 4 analyses with the Fisher Omnibus test are shown by "+" (for instance, the first row shows the results of statistical analysis for RM and ABI systems).

### Hypothesis: pAgo is a key component of a novel prokaryotic immune system in which it functions either as a nuclease or as a structural subunit of nuclease complexes that utilizes guide RNAs or DNAs to degrade virus/plasmid genomes

Several convergent lines of evidence point to defense against invading mobile elements as the primary function of pAgos. (1). The analogy to eukaryotic Argonautes many of which are dedicated to the defense against viruses and transposable elements. (2). The guide-DNA-dependent nuclease activity of AaAgo and TtAgo. (3). Extensive HGT of pAgos which is best compatible with a stress-response related function. (4). Preferential location of pAgo genes in genomic neighborhoods significantly enriched in known phage-defense genes. (5). Co-localization of PIWI-domain protein genes with genes encoding other (predicted) nucleases. (6). The near perfect complementarity between the predicted nuclease and guide-binding activities of pAgos and co-localization with other putative nucleases: the inactivated pAgos that lack the PAZ domain are associated with genes encoding predicted nucleases whereas the apparently active, PAZ-containing pAgos are not (Figure 3). The latter observation suggests that pAgos function within nuclease complexes, in some cases as their catalytic subunits, and in other cases, as structural subunits interacting with the actual nucleases.

**Figure 5:**
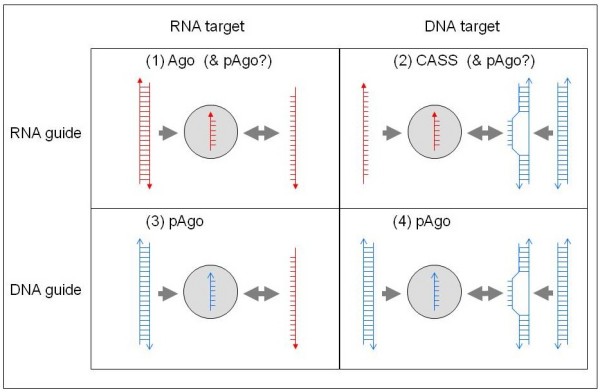
**Possible mechanisms of the hypothetical novel prokaryotic systems of defense against mobile elements centered around pAgo compared to the mechanisms of CASS and eukaryotic RNAi**. Currently, models (3) and/or (4) are the most likely functional mechanisms for pAgo (see text) but the eukaryotic Ago-like (1) and the prokaryotic CASS-like (2) models cannot be ruled out at this stage. RNA molecules are shown in red and DNA molecules in blue. Circles denote the proteins that form complexes with the guide RNA or DNA. Arrows indicate the directions of the respective processes.

Additional functional clues allow us to tentatively propose more specific mechanisms for the functions of pAgos in the defense of prokaryotes against mobile elements (Figure 5). In eukaryotic Argonautes, the PAZ domain binds the small guide RNA and facilitates its hybridization with the complementary region of the target mRNA. Most of the pAgos that are predicted to be active nucleases also contain PAZ domains suggesting that they function via a similar mechanism, in agreement with the experimental data for AaAgo and TtAgo [31,36,63,64]. The apparently inactivated pAgos lack PAZ domains but are co-localized with genes encoding predicted nucleases and the APAZ domain (Figure 1, 2). The (so far) exclusive presence of the APAZ domain within predicted operons encoding inactivated pAgos makes us speculate that, similary to PAZ domains, the APAZ domains bind guide molecules and target the putative nuclease complex to phage nucleic acids.

The PD-(D/E)XK superfamily nucleases, to which the predicted nucleases associated with the majority of pAgos are homologous, so far have been shown to cleave exclusively dsDNA. Thus, it seems most likely that the predicted pAgo-based defense systems directly target invader dsDNA genomes rather than mRNAs (Figure 5). On the other hand, as stated above, in vitro analyses have revealed that AaAgo and TtAgo are most active as DNA-guided ribonuclease, suggesting that RNA may be a target as well [REFS [35,36]]. The guide molecule could be either a small RNA (with the implication that the respective nuclease cleaves a RNA-DNA hybrid) or a small DNA as suggested by the study of AaAgo [63,64] and TtAgo [31,36].

The proposed model for the pAgo-based phage defense shows functional analogies to both CASS and the eukaryotic RNAi (Figure 5). Given the phylogenetic affinity of a distinct family of apparently active archaeal pAgos and eukaryotic Argonautes (Figure 3), this hypothetical defense system is the probable evolutionary progenitor of the eukaryotic RNAi. The spread of RNA viruses in eukaryotes that was accompanied by the displacement of the majority of DNA viruses [65] could have been the driving force behind the switch of the specificity of this defense system from DNA to RNA.

## Conclusion

The functions of the pAgos to some extent have been characterized *in vitro *(Yuan 2005)[31,36] but remain to be determined *in vivo*. The convergence of several lines of evidence discussed here seems to strongly support the hypothesis that pAgos are key components of a novel class of immune system that employ guide DNA or RNA molecules to destroy virus and plasmid DNA or mRNA). These proposed mechanisms of action suggest functional parallels between the predicted pAgo-based defense systems and CASS, and a direct evolutionary link between the former and eukaryotic RNAi. The predictions of the hypothesis, in particular, the nuclease activity catalyzed by PAZ-domain-containing but not by PAZ-domain-lacking pAgos, the complementary activities of associated putative nucleases, and guide DNA or RNA binding by the APAZ domains are amenable to straightforward experimental validation.

## Methods

### Sequence analysis

All analyzed sequences were from the non-redundant protein sequence database at the NCBI. Database searches were performed using PSI-BLAST [66], typically, with the inclusion threshold E = 0.01, and no composition-based statistics or low complexity filtering, or the HH search program available through the HHpred server [67]. Multiple alignments of protein sequences were constructed by combining the results obtained with the PROMALS program [68] and the MUSCLE program [69], followed by a minimal manual correction on the basis of local alignments obtained using PSI-BLAST [66]. Protein secondary structure was predicted using the PSIPRED program [70].

Maximum likelihood (ML) phylogenetic trees were constructed from the alignment of PIWI domain region (only positions with less than 30% gaps were used for reconstruction – 258 altogether), by using the MOLPHY program [71] with the JTT substitution matrix to perform local rearrangement of an original Fitch tree [72]. The MOLPHY program was also used to compute RELL bootstrap values.

### Fisher Omnibus test

Only 45 completely sequenced genomes were used for this analysis; the complete genome information was obtained from FTP of RefSeq database (;[73]). Proteins in these genomes were assigned to COGs using a modified COGNITOR program [74]. The target sets of phages defense proteins were obtained from the following sources: restriction-modification (RM) systems related protein from REBASE [75]; abortive infection (ABI) related genes from the Chopin et al. review [76]; CRISPR systems related genes from [22] and toxin-antitoxin related genes from [77]. Proteins of the RM and ABI systems were assigned to COG as indicated above, and for other systems, COG numbers have been already reported in the aforementioned papers (see the complete list of these COGs in Additional File 5).

In each genome, we identified the genes that belong to each of the aforementioned four well-characterized phage defense systems and computed the gene counts for each system in the entire genome (*K *phage defense genes in a genome containing *N *genes) as well as within each of windows of size ± *w *= 10 surrounding each pAgo gene (*k *genes in window). For each window, the probability to observe ≥*k *phage defense genes by chance was approximated using the binomial distribution:



The results obtained for multiple windows were combined using the Bailey and Gribskov's variant of the Fisher Omnibus test [63].



## Competing interests

The authors declare that they have no competing interests.

## Authors' contributions

KSM and JVDO initiated the study; KSM performed sequence analysis and genome comparison; YIW devised and performed the statistical tests; KSM, JVDO and EVK interpreted the results and formulated the hypothesis; KSM and YIW wrote the first draft of the manuscript; EVK and JVDO wrote the final manuscript that was read and approved by all authors.

## Reviewers' comments

### Reviewer 1

#### Daniel Haft, The J. Craig Venter Institute

Draft Public Comments

"Emerging evidence about prokaryotic homologs of Argonaute (pAgo) makes it clear that these proteins are related to their eukaryotic counterparts not just in sequence and structure, but also in molecular function. They might be related as well in terms of biological process, perhaps with many or most serving a primary function of phage resistance rather than of host gene transcriptional regulation. The case made in this manuscript, as argued by the interpretation of protein domain architecture, is highly suggestive. However, the statistical test for genomic association of pAgo with other phage resistance systems is currently unconvincing in the absence of a negative control. Other possible roles for pAgos seem equally consistent with available data."

#### Authors' response

*a negative control, namely, a test of the possible association of pAgos with mobile genes that are not involved in phage defense is included in the revised manuscript *(see Additional File 5). *As the result of this test was indeed negative, we find the statistical evidence as convincing as it can be although the final proof, of course, can only be experimental*.

"One alternate possibility is that most pAgos serve as machinery for boutique host regulatory systems. Anti-sense RNA expression in bacteria has been underappreciated; its prevalence likely is still underestimated. Some antisense RNA is cis-acting, through a mechanism of transcriptional interference, but some is trans-acting, through mechanisms of dsRNA formation. Since the trans-acting antisense RNAs themselves have won only a limiting understanding, it stands to reason that mechanisms acting downstream of dsRNA formation also are incompletely understood. A role for many pAgo proteins in the control of host gene expression seems quite likely."

#### Authors' response

*The possibility that some pAgos are also involved in regulation of bacterial genes is certainly interesting and not implausible. However, the data presented in this paper suggest to us that the functions in defense against mobile elements are primary*.

"A second possibility for these systems, supported by their apparent high degree of lateral transfer, is that most are selfish genetic elements. By analogy to transposons, homing endonucleases encoded within inteins, and temperate phage, these systems may carry out nuclease reactions simply to mediate their own spread. Some incidental benefit to host genomes is possible; any endogenous nuclease, it may be assumed, has some potential to cleave phage DNA or RNA, as in the example of ribonuclease HIII vs. RNA phage. But that level of phage resistance capability could be regarded as secondary."

#### Authors' response

*All prokaryotic defense and stress response systems are to a large extent selfish as discussed in detail for restriction-modification and toxin-atitoxin systems. We strongly suspect that this is indeed the case for the putative pAgo-centered system as well*.

"The extreme selective pressures of phage/host warfare make it quite likely that the proposed role for pAgos in phage resistance in prokaryotes is at least occasionally true. The greater question is whether pAgos proteins represent a new, major player in prokaryotic resistance to phage attack, and whether most pAgos proteins have host defense as a primary role. This is a mirror to the question of whether CRISPR arrays might be co-opted to serve perform regulatory functions, given their extreme plasticity and their transcription into small RNAs – one might examine repeat arrays in after phage-free serial passage of selected strains under extreme selection."

#### Authors' response

*Cooperation of pAgo with the CRISPR system cannot be ruled out but appears unlikely. Of the 780 bacterial and archaeal genomes that we analyzed for the presence of CRISPR and pAgo, 291 encoded CRISPR and 51 encoded pAgo, with the overlap of only 28 genomes. Of course, the localization of the pAgo gene within the Cas gene array in Methanopyrus kandleri is suggestive but so far this remains the only genome that shows such an association*.

"Restriction enzyme systems, especially restriction/modification systems, discriminate self vs. non-self by recognizing short sequence signatures in phage that are either masked or missing in the host. CRISPR systems discriminate self from non-self by capture and expression of samples of exogenous DNA. Both abortive infection systems and toxin-antitoxin systems have the potential to shut down the host cell, in response to stress from phage infection, in order to block the phage life cycle. Each of these schemes provides a clear model of how defense mechanisms are triggered. The trickiest part of the model for pAgos in phage defense concerns the source of guide DNA or RNA. Is it DNA encoded on the host chomosome? Will it have a promoter and a terminator? It seems at least theoretically possible that CRISPR arrays themselves might be a source. If a typical CRISPR system targets phage DNA according to exact matches to spacer sequences, one might postulate a backup system in which the same small RNAs, with some tolerance for mismatches, silence phage mRNA. It therefore makes sense to ask – what fraction of pAgos-containing genomes have CRISPR systems, and is the prevalence significantly higher for any subgroup of pAgos?"

#### Authors' response

*It is indeed true that we do not have any inkling of the source of the putative guide DNA or RNA that is employed by pAgo. The idea that pAgo might share the guide molecules with CRISPR is very interesting. The problem is that, as indicated above, there is no clear sign of cooperation between pAgo and CRISPR, and what is most damning for this provocative idea, is that the majority of the genomes that encode pAgo possess no CRISPR*.

*We attempted to search for sequence conservation and repetitive elements in the upstream and downstream regions of pAgo operons but failed to find anything suggestive. When more closely related genomes encoding pAgo become available, it will be necessary to repeat this attempt*.

A reasonable view of genome organization is that some regions of a genome are more plastic than others. The more plastic regions would be expected to accumulate prophages, transposons, integrated plasmids, conjugation regions, pseudogenes, and "fitness factors" such as CASS, antibiotic resistance genes, virulence genes, and capsular polysaccharide genes, all in close proximity. In this view, genes encoding restriction systems and CRISPR systems likely would occur close to each other because both the region tolerates insertion, not because both system mediate host defense. The statistical argument, therefore, does not currently allow one to discriminate phage defense from other possible functions for these systems. If the statistical association with RM and CASS is not replicated by associations with secretion systems, pilus proteins, integrases and recombinases, plasmid partition proteins, capsular polysaccharide biosynthesis genes, etc, then it may become somewhat more convincing.

#### Authors' response

*We appreciate this suggestion and sought to test the hypothesis that co-localization of pAgo genes with those for other systems of antiphage defence is a trivial consequence of the occurrence of all these genes in highly plastic regions of prokaryotic genomes. To this end, we examined the potential association of pAgo genes with typical components of the mobilome such as transposases, integrases, and various genes of apparent phage origin. As indicated in the revised text of the article and presented in detail in the *Additional Files 5 and 6, *there was no significant association between pAgo and the elements of the mobilome. Thus we believe that the most parsimonious interpretation of the data is that there are indeed phage defence islands in prokaryotic genomes and pAgo genes show a strong association with these islands*.

### Reviewer 2

#### Martijn Huynen, Radboud University, Nijmegen Medical Centre

The manuscript by Makarova and co-workers provides a compelling argument for the functional link between Bacterial and Archaeal Argonaute proteins and proteins that are involved in defense against "foreign" DNA.

I only have a few comments:

Studies on the value of the genomic association of genes for the prediction of functional links between proteins have gone to a great length to actually benchmark at which level of genomic association it not only becomes statistically significant, but also functionally meaningful in terms of predicting that proteins are actually involved in the same pathway. I cannot judge the level of "functional relevance" of the P-values provided in table 1.

Along the same lines: can the authors give simple numbers of how often the four protein families were discovered in the vicinity of the 100 pAgos genes.

#### Authors' response

*This information is now available in the new *Additional File 6*for the set of 45 genomes that were analyzed using the Fisher Omnibus test*.

I take it that all genomes that were included in the significance study were phylogenetically distant enough to assure that gene order conservation was not trivial?

#### Authors' response

*No, we did this analysis for all available genomes, since even in some closely related genomes the location of the pAgo operons is different. In response to these concerns, we have redone the analysis for distantly related genomes only. The results have not substantially change; actually, even more significant p-values were obtained *(see the new Additional File 6).

"This analysis resulted" I cannot find how this analysis was done, Fisher Ombnibus test mentioned in the methods does not require genes to be part of the same potential operon, and "predicted to be co-expressed" can thus not be concluded from it.

#### Authors' response

*In the revised manuscript, the criteria for calling potential operons are given explicitly*.

### Reviewer 3

#### Chris Ponting, Oxford University

Makarova et al. have undertaken a thorough and illuminating analysis of prokaryotic Argonaute homologs. Their analysis consists first of detailed sequence analysis of PIWI domain homologs followed by investigation of putative operons. The manuscript ends with a nice demonstration that pAgo genomic regions are significantly enriched for phage defense genes. This allows them to pose an important and testable hypothesis which provides the major contribution of this paper. The manuscript is well written and its analyses are sound.

## Supplementary Material

Additional file 1 **The list of all identified PIWI domain containing proteins and their closest neighborhood**. The data provided represent list of all identified PIWI domain containing proteins that were further analyzed in this work. Click here for file

Additional file 2 **Multiple alignment for full length PIWI domain containing proteins**. The provided alignment shows distinct group of PIWI proteins. Click here for file

Additional file 3 **Multiple alignment of uncharacterized C-terminal domain of proteins also containing N-terminal nuclease domain and associated with PIWI proteins**. The provided alignment shows the previously undetected domain associated with PIWI proteins. Click here for file

Additional file 4 **Multiple alignment of uncharacterized N-terminal domain of proteins also containing C-terminal nuclease domain and associated with PIWI proteins**. The provided alignment shows the previously undetected domain associated with PIWI proteins. Click here for file

Additional file 5 **The list of all COGs implicated in antiphage defense**. The data provided represent list of phage defense COGs of four distinct systems used for the Fisher Omnibus test. Click here for file

Additional file 6 **The data used for the Fisher Omnibus test**. The file contains data and calculations for the Fisher Omnibus test. Each worksheet corresponds to the analysis of a distinct set of phage defense COGs (see also AF3_Ph_def_COGs.xls). On the left hand side are calculations for the whole set of genome. On the right hand side, highlighted in yellow, calculations for a representative set of genomes (closely related genomes were excluded). Click here for file
